# Semi-Automated Neuromelanin-Sensitive MRI Reveals Substantia Nigra Volume Reduction in Early Parkinson’s Disease with Moderate Diagnostic Performance

**DOI:** 10.3390/diagnostics16071046

**Published:** 2026-03-30

**Authors:** Arturs Silovs, Gvido Karlis Skuburs, Nauris Zdanovskis, Aleksejs Sevcenko, Janis Mednieks, Edgars Naudins, Santa Bartusevica, Solvita Umbrasko, Liga Zarina, Laura Zelge, Agnese Anna Pastare, Jelena Steinberga, Jurgis Skilters, Baingio Pinna, Ardis Platkajis

**Affiliations:** 1 Department of Radiology, Riga East University Hospital, Hipokrata Street 2, LV-1038 Riga, Latvia; nauris.zdanovskis@rsu.lv; 2 Department of Radiology, Riga Stradins University, Dzirciema Street 16, LV-1007 Riga, Latvia; gvido.skuburs@gmail.com (G.K.S.); edgars.naudins@gmail.com (E.N.); ardis.platkajis@rsu.lv (A.P.); 3 Latvian Maritime Medicine Centre, Patversmes Street 29, LV-1005 Riga, Latvia; 4 Department of Neurology, Riga Stradins University, Dzirciema Street 16, LV-1007 Riga, Latvia; aleksejs.sevcenko@inbox.lv (A.S.); janis.mednieks@gmail.com (J.M.); 5 Laboratory for Perceptual and Cognitive Systems, University of Latvia, Raiņa Bulvāris 19, LV-1586 Riga, Latvia; santa.bartusevica@lu.lv (S.B.); solvita.umbrasko@lu.lv (S.U.); liga.zarina@lu.lv (L.Z.); laura.zelge32@gmail.com (L.Z.); pastare.a.a@gmail.com (A.A.P.); je.petersone@gmail.com (J.S.); jurgis.skilters@lu.lv (J.S.); 6 Department of Biomedical Sciences, University of Sassari, 07100 Sassari, Italy; baingio@uniss.it

**Keywords:** Parkinson’s disease, neuromelanin, substantia nigra, magnetic resonance imaging, biomarker, semi-automated analysis, volumetric measurement

## Abstract

**Background:** Parkinson’s disease (PD) is characterized by progressive degeneration of dopaminergic neurons in the substantia nigra pars compacta, accompanied by neuromelanin loss. Neuromelanin-sensitive magnetic resonance imaging (NM-MRI) enables in vivo visualization of these changes; however, its diagnostic and clinical utility remains incompletely defined. This study evaluated the feasibility, reliability, and biological sensitivity of semi-automated NM-MRI–based substantia nigra volumetry in PD. **Methods:** In this prospective case–control study, 50 participants (25 PD patients and 25 healthy controls) underwent 3T NM-sensitive MRI using a high-resolution T1-weighted spin-echo sequence. Semi-automated segmentation of hyperintense substantia nigra regions was performed using Mango v3.5.1, with intracranial volume normalization derived from FreeSurfer v7.3. Four participants were excluded due to motion artifacts, yielding a final cohort of 46 subjects. Clinical assessment included the Unified Parkinson’s Disease Rating Scale (UPDRS) Part III and Hoehn and Yahr (H&Y) staging. Group comparisons, receiver operating characteristic (ROC) analysis, and reliability testing using intraclass correlation coefficients (ICC) were performed. **Results:** Corrected substantia nigra volume was significantly reduced in PD patients compared with controls (18% reduction; *p* = 0.039, Mann–Whitney U test). Semi-automated measurements demonstrated excellent agreement with manual segmentation (ICC = 0.945). ROC analysis showed moderate discriminative performance for corrected volume (AUC = 0.700; sensitivity 68.4%, specificity 74.1%). No significant correlation was observed between corrected substantia nigra volume and UPDRS-III motor scores, while a trend toward lower SNc volume was observed with advancing H&Y stage. **Conclusions:** Semi-automated NM-MRI volumetry detects biologically meaningful substantia nigra volume loss in early-stage Parkinson’s disease with high measurement reliability. However, diagnostic performance was moderate and insufficient for standalone clinical diagnosis or motor severity prediction. These findings support the role of NM-MRI as a complementary imaging marker within multimodal diagnostic and research frameworks rather than as an independent diagnostic tool.

## 1. Introduction

Parkinson’s disease (PD) affects approximately 1–2 per 1000 individuals globally, with prevalence doubling in recent decades and projected to reach 12.9 million patients by 2040 [1]. Despite its widespread impact on public health and quality of life, early and accurate PD diagnosis remains challenging, as characteristic brain changes begin more than 10 years before clinical symptom onset [2]. This prodromal phase, combined with symptom overlap with other Parkinsonian syndromes such as multiple system atrophy (MSA), significantly complicates differential diagnosis and often leads to diagnostic errors [3,4].

The pathological hallmark of PD is the progressive degeneration of dopaminergic neurons in the substantia nigra pars compacta (SNc), which contains neuromelanin (NM), a dark pigment closely associated with disease pathogenesis [3]. The SN, located in the midbrain, is a primary site of pathology in PD. In this manuscript, “SN” refers to the entire substantia nigra region, while “SNc” is used when specifically referring to the dopaminergic pars compacta. Under normal conditions, the dopaminergic neurons of the SN pars compacta produce NM as a byproduct of catecholamine metabolism [4]. NM accumulates gradually with age in these neurons, giving the SN its characteristic black color.

In PD, NM levels decrease due to dopaminergic neuronal degeneration, and quantification of these changes could provide valuable additional information for both diagnosis and disease monitoring.

**Figure 1:**
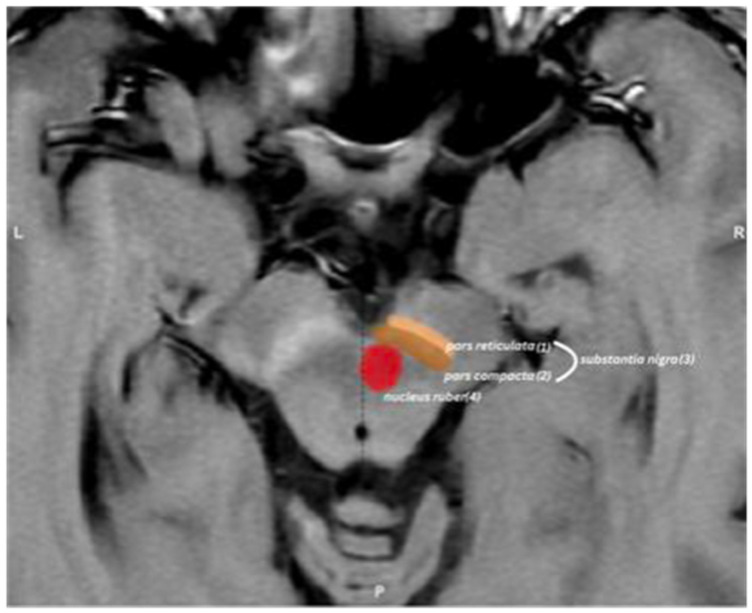
Neuromelanin visualization with NM-MRI, using adjusted T1-weighted imaging sequence: (1) Pars reticulata; (2) Pars compacta; (3) Substantia nigra; (4) Nucleus ruber.

Magnetic resonance imaging (MRI) offers several non-invasive methods for evaluating these changes. T1-weighted NM-sensitive sequences allow direct visualization of NM content in the SN region (Figure 1), while quantitative susceptibility mapping (QSM) provides information on iron deposition in brain structures [5], and diffusion tensor imaging (DTI) enables assessment of white matter tract connections between neurons [6]. Region-specific iron distribution changes have been documented in early idiopathic PD using quantitative susceptibility mapping [7]. Among these techniques, neuromelanin-sensitive MRI (NM-MRI) has emerged as a particularly promising approach, as it can directly detect NM-related signal changes that correlate with neuronal loss in PD [8,9,10,11].

The dual nature of neuromelanin presents both opportunities and challenges for PD research. Under normal conditions, NM appears to function as a neuroprotective agent by binding and neutralizing reactive oxygen species and sequestering transition metals such as iron, copper, zinc, and manganese [12]. However, under pathological conditions, NM can become toxic and contribute to neurodegeneration through several mechanisms [13], including serving as a pro-oxidant when iron-NM balance is disrupted [14,15,16], activating microglia when released from dying neurons [17], and releasing accumulated toxins during neuronal death [18,19]. This dual biological behavior is directly relevant to NM-MRI signal generation, as neuromelanin-associated paramagnetic and macromolecular properties influence T1 shortening and hyperintensity, thereby linking cellular pathology with imaging-derived biomarkers.

Recent advances in high-resolution MRI and automated image analysis techniques have improved the precision and reproducibility of NM measurements, making them more suitable for clinical applications. Semi-automated segmentation methods can reduce operator bias while maintaining accuracy, potentially facilitating wider adoption of NM-MRI in clinical practice [20].

The objective of this study was to evaluate the clinical association between NM-MRI–derived substantia nigra volumetric changes and motor symptoms in PD patients using high-resolution NM-sensitive MRI with semi-automated image analysis. We hypothesized that: (1) NM-sensitive MRI can detect substantia nigra volumetric alterations; (2) these NM-MRI-derived SN volumes can be accurately measured using semi-automated software; and (3) these imaging-derived volumetric measures may show associations with clinical disease severity.

## 2. Materials and Methods

### 2.1. Study Design and Participants

This prospective case–control study was conducted between November 2023 and February 2025. Patients were recruited from multiple neurology outpatient clinics, while all MRI examinations were performed in a single tertiary radiology department using one 3T scanner to ensure standardized image acquisition.

The study protocol was approved by the institutional ethics committee, and all participants provided written informed consent prior to inclusion.

Inclusion criteria for Parkinson’s disease patients were: clinically confirmed diagnosis of idiopathic Parkinson’s disease according to Movement Disorder Society criteria, age 18–70 years, and ability to undergo MRI examination. Healthy controls were required to have no history of neurological or psychiatric disease and no clinical signs of Parkinsonism. Exclusion criteria for all participants included prior stroke, intracranial tumors, central nervous system inflammation, moderate or severe traumatic brain injury, significant metabolic or systemic disease affecting brain structure, and MRI artifacts precluding reliable image analysis. Groups were matched for sex distribution. All clinical assessments were performed in the ON-medication state to reflect routine clinical conditions. Disease duration was recorded for all patients; however, due to limited variability, it was not included as a primary covariate.

Although idiopathic Parkinson’s disease most commonly presents after the age of 60, younger patients were included to capture early-onset phenotypes. However, no patients with atypical Parkinsonism were included. The resulting age imbalance is acknowledged as a limitation and addressed through adjusted statistical analysis.

### 2.2. Clinical Assessment

All Parkinson’s disease patients underwent standardized clinical assessment performed by experienced neurologists. Motor symptoms were evaluated using the Unified Parkinson’s Disease Rating Scale (UPDRS), including Part III [21,22]. Clinical staging was determined using the modified Hoehn and Yahr (H&Y) scale (Table 1).

Clinical evaluations were performed in the ON-medication state, reflecting routine outpatient clinical practice. Disease duration at the time of imaging was recorded; however, due to limited variability within the cohort, it was not used as a primary stratification variable.

The ranges shown reflect typical clinical distributions rather than validated conversion thresholds (Table 2).

### 2.3. MRI Acquisition

All MRI examinations were performed on a single 3T scanner (SIGNA™ Pioneer 3T wide bore MRI, GE Healthcare, Chicago, IL, USA) using a standardized protocol, identical hardware and imaging parameters, minimizing inter-scan variability. The NM-sensitive sequence was a high-resolution T1-weighted spin-echo sequence with the following parameters:

- Repetition time (TR): 600 ms;
- Echo time (TE): 10 ms;
- Field of view (FOV): 200 mm;
- Matrix size: 512 × 320;
- Pixel size: 0.43 × 0.69 mm;
- Slice thickness: 2.5 mm;
- Inter-slice gap: 1 mm;
- Number of slices: 15;
- Acquisition time: 6 min 27 s.

The imaging volume extended from the corpus callosum to the inferior border of the pons, ensuring complete coverage of the substantia nigra region [8].

### 2.4. Image Analysis

#### 2.4.1. Semi-Automated Segmentation

Image analysis was performed using Mango v3.5.1 software with a semi-automated segmentation approach. Four participants (two controls and two PD patients) were excluded due to motion artifacts preventing reliable measurements.

A standardized workflow was applied to all participants. First, a multiplication factor (MF) was derived from a subset of control subjects to normalize substantia nigra signal intensity relative to mean midbrain intensity (MMI). Manual delineation of the substantia nigra was independently performed by two neuroradiologists with more than five years of experience to establish reference measurements.

**Figure 2:**
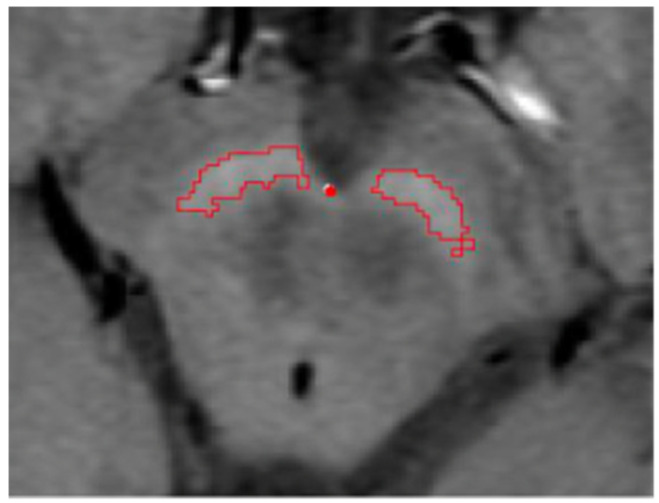
Automated hyperintense SN region segmentation and delineation based on MMI and MF.

Individual intensity thresholds were then calculated for each participant, enabling automated segmentation of hyperintense SN voxels on NM-sensitive MRI (Figure 2). Total segmented volume was normalized to intracranial volume to account for interindividual differences in head size. In this study, the measured volumetric variable represents NM-MRI–derived hyperintense substantia nigra volume, used as an imaging proxy for neuromelanin-related nigral integrity rather than a direct histological measure of neuromelanin.

Volume Calculation and Normalization: Total neuromelanin-related signal volume was calculated and normalized to intracranial volume (ICV) using FreeSurfer 7.3 software.

#### 2.4.2. Mean Midbrain Intensity Quantification

**Figure 3:**
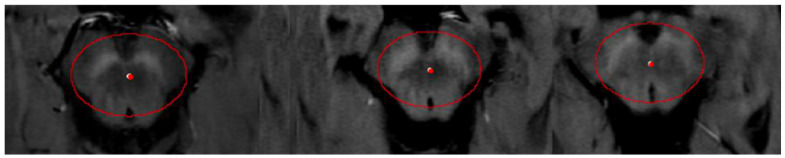
Mean Midbrain intensity quantification using three consequent regions.

To normalize signal intensity across participants, mean midbrain intensity was calculated using three oval regions of interest placed on three consecutive axial slices at the level of the midbrain, starting from the first visible boundary between the mammillary bodies and cerebral peduncles (Figure 3). ROI size was adapted to individual anatomy while maintaining consistent anatomical landmarks across participants. Although limited slice sampling may introduce variability, this approach reflects previously published NM-MRI methodologies [20] and was supported by high inter-method reliability in this study.

Although this approach samples a limited anatomical volume, it was applied consistently across all participants. Potential variability introduced by this strategy was mitigated by subsequent normalization procedures and by assessment of inter-method reliability.

#### 2.4.3. Volume Measurement

NM-MRI-derived substantia nigra volume was quantified by outlining oval regions encompassing the visually identified substantia nigra across three or more consecutive slices. While region size was adapted to individual anatomy, identical anatomical landmarks and segmentation rules were applied across participants to ensure consistency. The total SN volume was calculated as:

SN Volume = NΣ(Area) × h

where NΣ(Area) represents the sum of SN areas across all measured slices, and h represents slice thickness.

#### 2.4.4. Volume Correction

Corrected NM-MRI-derived substantia nigra volume (Cvol) was calculated to normalize for individual head size differences, using following formula:

$$Cvol=\frac{SN\;Volume\left({mm}^{3}\right)}{eTIV({mm}^{3})}$$

where eTIV is total intracranial volume, estimated using FreeSurfer v7.3 whole brain segmentation and summing white matter, gray matter, and cerebrospinal fluid volumes.

#### 2.4.5. FreeSurfer Whole Brain Segmentation and Intracranial Volume Estimation

To obtain accurate intracranial volume (ICV) measurements for normalization of substantia nigra volumes, we employed FreeSurfer v7.3 (Laboratory for Computational Neuroimaging, Athinoula A. Martinos Center for Biomedical Imaging, Charlestown, MA, USA) for automated whole-brain segmentation and volumetric analysis. FreeSurfer is a widely validated neuroimaging analysis suite that provides reliable and reproducible measurements of brain structure volumes [23,24,25].

The FreeSurfer processing pipeline was applied to the high-resolution 3D T1-weighted MPRAGE images acquired for each participant. The automated processing workflow consisted of several key steps:

1. Preprocessing and Skull Stripping: Raw T1-weighted images underwent motion correction, intensity normalization, and skull stripping to remove non-brain tissue.
2. Tissue Classification and Segmentation: Following preprocessing, the brain volume was segmented into distinct tissue types using a probabilistic atlas-based approach.
3. Quality Control and Manual Review: All FreeSurfer outputs underwent visual quality control assessment to identify potential segmentation errors.
4. Intracranial Volume Calculation: The estimated total intracranial volume (eTIV) was calculated as the sum of all segmented brain tissues (cortical gray matter, subcortical gray matter, white matter) plus cerebrospinal fluid spaces within the cranial cavity. This measurement provides a proxy for premorbid brain size and is commonly used for normalizing regional brain volumes to account for individual differences in head size. The eTIV calculation follows the formula:
  eTIV = Total Cortical Gray Matter + Subcortical Gray Matter + Cerebral White Matter + Cerebrospinal Fluid

### 2.5. Statistical Analysis

Statistical analyses were performed using SPSS Statistics (version 21, IBM Corp., Armonk, NY, USA). Normality of distributions was assessed visually and using the Shapiro–Wilk test. Continuous variables are presented as mean ± standard deviation.

Between-group comparisons were performed using independent-samples *t*-tests or Mann–Whitney U tests as appropriate. Receiver operating characteristic (ROC) analysis was used to assess discriminative performance, with area under the curve (AUC) values interpreted as measures of diagnostic accuracy. Optimal thresholds were determined using Youden’s index.

Reliability between semi-automated and manual measurements was assessed using intraclass correlation coefficients (ICC; absolute agreement model). Correlations between imaging metrics and clinical scores were evaluated using Spearman or Pearson correlation coefficients as appropriate. Statistical significance was set at *p* < 0.05.

## 3. Results

### 3.1. Participant Characteristics

**Figure 4:**
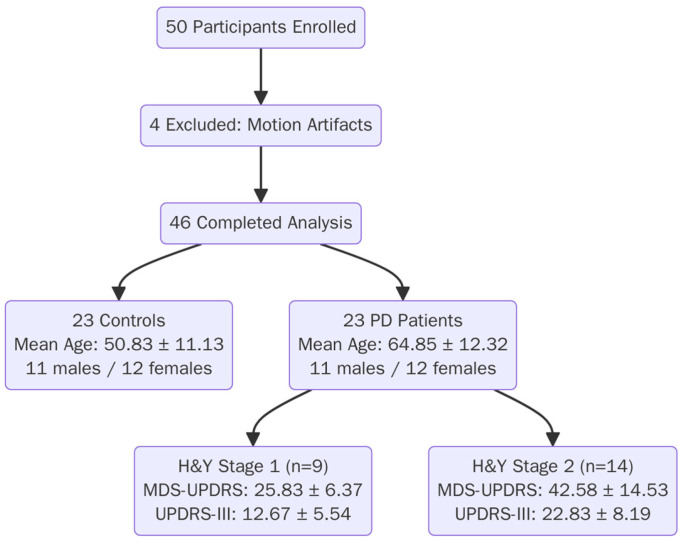
Patient enrollment scheme with clinical stage distribution.

A total of 50 participants were enrolled, with 46 completing the analysis after excluding 4 participants due to motion artifacts. The final cohort included 23 controls (mean age 50.83 ± 11.13 years; 11 males/12 females) and 23 PD patients (mean age 64.85 ± 12.32 years; 11 males/12 females) (Figure 4).

PD patients were stratified by H&Y stage: H&Y Stage 1 (*n* = 9, MDS-UPDRS Parts I–III 25.83 ± 6.37, MDS-UPDRS III 12.67 ± 5.54) and H&Y Stage 2 (*n* = 14, MDS-UPDRS Parts I–III 42.58 ± 14.53, UPDRS-III 22.83 ± 8.19). Statistical analysis revealed significant differences in UPDRS-III scores between H&Y stages (*p* = 0.009, Mann–Whitney U test), confirming significant differences in motor symptoms between the groups. Participant characteristics are summarised in Table 3.

### 3.2. Neuromelanin Volume Measurements

NM-MRI parameters showed trends toward reduced SN area and volume in PD patients [26] compared to controls, though these differences did not reach statistical significance (*p* > 0.05) (Table 4). Similarly, separate analysis of left and right hemispheres revealed no statistically significant differences between patients and controls for uncorrected measurements.

**Figure 5:**
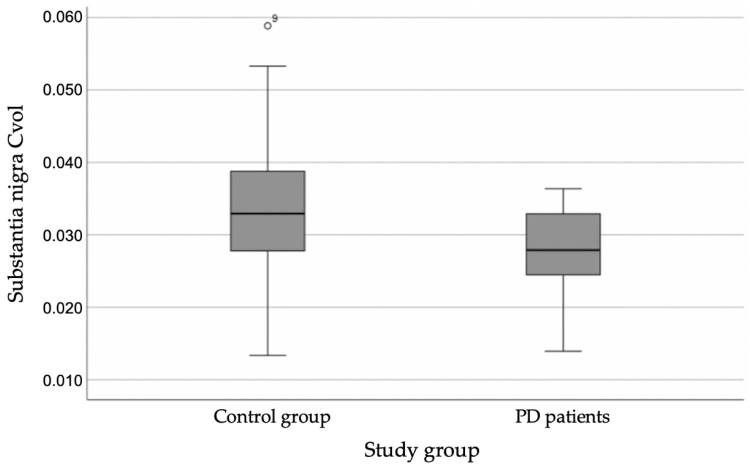
Corrected Substantia nigra volume difference between study groups.

After correction for intracranial volume, significant differences were found between SN volumes. The corrected SN volume (Cvol) was significantly reduced in PD patients compared to controls:

Controls demonstrated a corrected SN volume of 0.0337 ± 0.0102, whereas Parkinson’s disease patients demonstrated a mean corrected SN volume of 0.0276 ± 0.0058. This corresponded to an absolute mean difference of 0.0061 mm^3^ (95% CI 0.0013–0.0109), representing an approximate 18% reduction in the PD group compared with controls (Figure 5). The between-group difference reached statistical significance (*p* = 0.039, Mann–Whitney U test).

To account for the observed age imbalance between groups, an analysis of covariance (ANCOVA) was performed with corrected SN volume as the dependent variable, group as the fixed factor, and age and sex as covariates. The group effect remained statistically significant after adjustment (F(1, 42) = 5.62, *p* = 0.022, partial η^2^ = 0.118), indicating that the observed reduction in substantia nigra volume in Parkinson’s disease patients is not solely attributable to age-related differences.

Additionally, we analyzed the association between MDS-UPDRS-III scores and corrected SN volume. There were no statistically significant findings on either SN side (Table 5). Linear regression confirmed that Cvol (R. and L.) did not predict MDS-UPDRS-III scores (R^2^ = 0.011, *p* = 0.668).

### 3.3. Diagnostic Performance

Receiver operating characteristic (ROC) analysis demonstrated moderate diagnostic performance for corrected SN volume in distinguishing Parkinson’s disease patients from controls (AUC = 0.700, 95% CI 0.549–0.851; *p* = 0.022). The optimal threshold value of 0.0269 yielded a sensitivity of 68.4% and a specificity of 74.1%, corresponding to a false positive rate of 25.9%.

**Figure 6:**
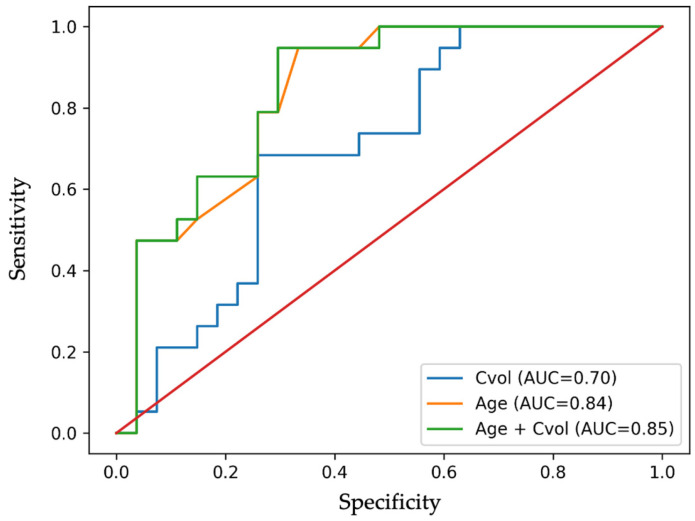
Receiver operating characteristic (ROC) curves comparing the discriminative performance of corrected SN volume (Cvol), age, and the combined model.

Given the age imbalance between groups, additional analyses were performed to assess the influence of demographic factors on classification performance. Age alone demonstrated strong discriminative ability (AUC = 0.84), reflecting group differences in this cohort. The combined model incorporating both age and corrected SN volume showed a modest improvement in classification performance (AUC = 0.85), indicating that NM-MRI-derived volumetry provides additional discriminatory information beyond age (Figure 6).

These findings support the interpretation of neuromelanin-sensitive MRI as a complementary imaging marker rather than a standalone diagnostic tool.

### 3.4. Clinical Correlations

No significant correlations were observed between MDS-UPDRS-III scores and corrected SN volume in either hemisphere. For the right hemisphere, Spearman ρ = −0.225 (*p* = 0.368), and for the left hemisphere, Spearman ρ = −0.119 (*p* = 0.626). Similarly, no significant correlation was found between total MDS-UPDRS Part I-III scores and corrected SN volume (ρ = −0.119, *p* = 0.626).

### 3.5. Disease Stage Analysis

SN volume was significantly reduced in both H&Y stage 1 and stage 2 patients compared with controls, without a clear separation between early disease stages. Controls demonstrated a corrected SN volume of 0.0337 ± 0.0102, whereas H&Y stage 1 patients showed 0.0285 ± 0.0054 mm^3^ (*p* = 0.017) and H&Y stage 2 patients 0.0279 ± 0.0064 mm^3^ (*p* = 0.015), indicating statistically significant differences from controls [27].

### 3.6. Method Validation

Two neuroradiologists were asked to manually segment SN in all study group patients. To evaluate the reliability of the semi-automated NM segmentation method, comparisons between manual and automated measurements were performed using the intraclass correlation coefficient (ICC). ICC was calculated using an absolute agreement model appropriate for comparing different methods. The resulting ICC value of 0.945 indicates excellent agreement between both measurement [19] approaches. Graphical representation also demonstrates that data points closely align with the ideal line y = x, reflecting complete measurement concordance.

Mean squares analysis revealed:

- MS_subject = 34,529.67;
- MS_rater = 12,031.98;
- MS_res = 82.90.

These results confirm that measurement variability primarily originated from between-subject differences rather than methodological errors, validating the reliability of the semi-automated approach.

## 4. Discussion

In this study, we evaluated the feasibility, reliability, and biological sensitivity of semi-automated neuromelanin-sensitive MRI for quantifying substantia nigra volume in patients with early-stage Parkinson’s disease. Our results demonstrate a significant reduction in corrected SN volume in Parkinson’s disease compared with healthy controls, accompanied by excellent agreement between semi-automated and manual measurements. However, diagnostic performance was moderate, and no significant association was observed between substantia nigra volume and MDS-UPDRS-III motor severity.

These findings indicate that while neuromelanin-sensitive MRI captures biologically meaningful structural changes in the substantia nigra, it does not provide sufficient accuracy for standalone diagnosis or motor severity assessment. Instead, NM-MRI volumetry should be regarded as a complementary imaging marker within multimodal diagnostic and research frameworks. Importantly, the present findings do not negate the biological relevance of NM-MRI but rather refine its role as a quantitative research and complementary imaging biomarker rather than a standalone diagnostic tool.

### 4.1. Interpretation of Substantia Nigra Volume Reduction

The observed 18% reduction in corrected SN volume in Parkinson’s disease patients is consistent with postmortem and in vivo imaging studies demonstrating progressive neuromelanin loss due to dopaminergic neuronal degeneration [19,27]. Importantly, volume differences reached statistical significance only after normalization to intracranial volume, underscoring the importance of head-size correction in quantitative nigral assessments. However, the borderline statistical significance observed (*p* = 0.039) should be interpreted cautiously given the sample size, and replication in larger, age-matched cohorts is required.

The absence of statistically significant differences in uncorrected SN volume measures suggests that raw neuromelanin-derived metrics may lack sufficient sensitivity when interindividual anatomical variability is not accounted for. This finding highlights a methodological consideration that may partially explain inconsistencies across prior NM-MRI studies and supports the use of normalization strategies in future work [28].

### 4.2. Diagnostic Performance and Clinical Correlations

Receiver operating characteristic analysis demonstrated moderate discriminative performance of corrected SN volume (AUC = 0.700), indicating limited utility as a standalone diagnostic test. This is consistent with prior meta-analyses reporting pooled AUC values ranging from 0.72 to 0.85 depending on imaging protocol and patient selection [29,30].

Importantly, age alone demonstrated substantially higher discriminative performance (AUC = 0.84), reflecting the age imbalance within the cohort.

The combined model incorporating both age and corrected SN volume resulted in only a minimal improvement in diagnostic accuracy (AUC = 0.85; ΔAUC = 0.01), suggesting that the primary discriminative contribution in this cohort is driven by age rather than NM-MRI-derived volumetric measures.

These findings indicate that while neuromelanin-sensitive MRI captures biologically meaningful structural changes, its incremental diagnostic value beyond basic demographic variables appears limited in this dataset.

Notably, despite the strong discriminative performance of age in ROC analysis, age was not a significant predictor of substantia nigra volume in the ANCOVA model, and group differences remained significant after adjustment. This suggests that neuromelanin-related structural changes are not solely attributable to age and likely reflect disease-specific neurodegeneration.

### 4.3. Disease Stage Considerations

Stratification by Hoehn and Yahr stage revealed reduced substantia nigra volumes in both stage 1 and stage 2 Parkinson’s disease patients compared with controls. While differences between early stages were modest, this pattern suggests that neuromelanin-sensitive MRI is capable of detecting nigral degeneration even at relatively early clinical stages.

The limited separation between stage 1 and stage 2 volumes indicates that cross-sectional NM-MRI volumetry may be insufficient for fine-grained staging within early disease phases. Longitudinal imaging studies will be required to determine whether individual trajectories of neuromelanin loss provide greater sensitivity to disease progression than single time-point measurements.

### 4.4. Methodological Strengths and Reproducibility

A major strength of this study is the high reproducibility of semi-automated substantia nigra segmentation, demonstrated by excellent agreement with manual measurements (ICC = 0.945). This finding supports the technical validity of the proposed workflow and suggests that semi-automated approaches may reduce operator-dependent variability inherent in manual segmentation.

All imaging was performed using a single MRI scanner and standardized acquisition parameters, minimizing inter-scan variability. While the segmentation approach relied on limited slice sampling and anatomically adapted regions of interest, consistent application across participants and strong inter-method reliability mitigate concerns regarding measurement instability. Although semi-automated segmentation currently requires dedicated software, similar workflows are increasingly being integrated into clinical radiology platforms, suggesting potential future clinical applicability.

### 4.5. Limitations

Several limitations should be considered when interpreting these findings. Most notably, the substantial age difference between control and Parkinson’s disease groups represents a critical confounding factor that could partially explain the observed volume differences independent of disease pathology. Neuromelanin accumulates progressively with normal aging, typically increasing until the 6–7th decade of life before potentially plateauing or declining.

Therefore, older healthy individuals would be expected to have larger NM volumes than younger controls, which would be expected to bias results toward underestimating group differences [31]. The fact that we still observed significantly smaller volumes in the (older) PD group suggests our findings are robust, but this age discrepancy limits generalizability.

Several strategies could address this limitation in future studies: (1) strict age-matching within ±5 years, (2) statistical covariate adjustment for age using ANCOVA, (3) age-stratified analysis, or (4) establishing normative age-specific reference ranges. Post hoc age-adjusted analyses suggested persistence of group differences, although effect sizes were reduced.

The gender imbalance (controls: 12M/13F vs. PD: 12M/12F) is less problematic, as prior studies show minimal sex differences in neuromelanin content [32]. However, the smaller intracranial volumes typically observed in females underscore the importance of our ICV normalization approach.

### 4.6. Implications for Multimodal Imaging Approaches

Given the limited incremental diagnostic value observed in this study, neuromelanin-sensitive MRI is best interpreted as a complementary biomarker reflecting underlying neurodegeneration rather than a primary diagnostic discriminator, particularly in datasets with demographic imbalances. Integration with other MRI-based biomarkers, such as quantitative susceptibility mapping for iron deposition or diffusion-based measures of nigrostriatal connectivity, may improve overall diagnostic accuracy [33]

Multimodal imaging approaches that combine structural, microstructural, and biochemical information are increasingly recognized as necessary for capturing the complex pathophysiology of Parkinson’s disease, particularly in early or prodromal stages [34].

## 5. Conclusions

Semi-automated neuromelanin-sensitive MRI enables reliable detection of substantia nigra volume loss in early Parkinson’s disease and demonstrates excellent measurement reproducibility. However, diagnostic performance is moderate and insufficient for standalone clinical diagnosis or motor severity assessment.

These findings support the use of neuromelanin-sensitive MRI as a complementary imaging marker within multimodal diagnostic and research frameworks, particularly for studying nigral degeneration and validating imaging methodologies in Parkinson’s disease.

## Figures and Tables

**Table 1:** Hoehn and Yahr scale severity staging.

Stage	Hoehn and Yahr Scale Description
1	Unilateral involvement only usually with minimal or no functional disability
2	Bilateral or midline involvement without impairment of balance
3	Bilateral disease: mild-to-moderate disability with impaired postural reflexes; physically independent
4	Severely disabling disease; still able to walk or stand unassisted
5	Confinement to bed or wheelchair unless aided

**Table 2:** Modified H&Y and MDS-UPDRS Parts I–III scale relevance.

HYS	UPDRS		
	II	III	II + III
1	1–10	1–20	2–30
2	5–20	10–30	15–50
3	10–25	20–40	30–65
4	15–35	25–50	40–85
5	20–52	35–56	55–108

**Table 3:** Demographic and clinical data of the study group.

Study group demographic and clinical data		
Characteristics	Control	PD
Number of participants	23	23
Age (years)	50.83 ± 11.13	64.85 ± 12.32
Gender (Male/Female)	11/12	11/12
Clinical Group distribution		
MDS-UPDRS I–III	-	38.85 ± 15.70
MDS-UPDRS Part III	-	20.05 ± 9.04
H&Y	-	1.75 ± 0.55

**Table 4:** Substantia nigra area and volume difference between control and study groups.

NM Parameter	Control (*n* = 23)	PD (*n* = 23)	*p*
**SN Right (R) and Left (L). Area, mm^2^**	193.22 ± 54.37	168.24 ± 35.45	0.086
R	106.50 ± 28.07	98.44 ± 25.88	0.328
L	87.02 ± 25.07	73.83 ± 20.70	0.066
**SN R and L Volume, mm^3^**	483.06 ± 135.92	420.61 ± 88.62	0.086
R	266.25 ± 70.17	246.10 ± 64.70	0.328
L	217.54 ± 62.68	184.59 ± 51.75	0.088

**Table 5:** UPDRS III score and Substantia nigra corrected SN volume association.

Side	Correlation	Coefficient	*p* Value
Right	Spearman ρ	−0.225	0.368
Left	Spearman ρ	−0.119	0.626

## Data Availability

The data presented in this study are available from the corresponding author upon reasonable request due to ethical and privacy restrictions.
